# Medical Items

**Published:** 1916-09

**Authors:** 


### MEDICAL ITEMS

### SILVOL: A NOTABLE GERMICIDE.

For application to mucous surfaces as a germicide, silver nitrate has long been recognized as a distinctly meritorious agent. It has had one serious drawback, however—its use in solution frequently caused irritation. Finally, as was to have been expected, the art of the chemist has overcome this objection. The combination of silver with a proteid base robs the former of its irritating effect. At the same time there is no loss of antiseptic value.

A proteid-silver preparation that is meeting with marked favor by eye, ear, nose and throat specialists, as well as by specialists in genito-urinary diseases, is offered by Parke, Davis & Co. under the name of Silvol. That this product has a number of advantages over most of the silver salts hitherto used is evident from the numerous commendatory references to it that are finding their way into the medical press. An article in point has just come under the eye of the writer and is worth noting in this connection. It appears in the December issue of the Journal of Ophthalmology and Oto-Laryngology and is from the pen of William C. White, D.D.S., Ph.G., M.D., of the University of Louisville.

Dr. White describes Silvol as "a metallic silver in colloidal combination with a proteid base, and slightly alkaloidal in reaction. It occurs in black, metallic, lustrous scale, slightly hygroscopic and very readily soluble in water. In solution it gives a rich seal brown color and produces only a temporary stain to clothing or dressing, which is completely removed by rinsing in warm soap suds. The preparation is so soluble that it requires only a moment to make the necessary solution. It is practically non-irritating in any reasonable dilution. The solution does not require filtering; and I wish to emphasize this fact, as it has been my experience with other similar products, especially in heavy solution, that a tarry substance will appear upon the surface, and, unless this is filtered out, it produces more or less irritation to the sensitive mucous membranes upon drying, leaving a very disagreeable burning or smarting sensation to the parts. None of my patients complained of pain or showed any irritation when a ten-per-cent. solution was used; on the contrary, they have expressed a feeling of comfort and a soothing sensation immediately following the application."

In summarizing, Dr. White names these advantages as applying to Silvol: "Quick solubility in any solution necessary for application to mucous membrane; less staining than by other proteid silver preparations; high percentage of silver content; minimum amount of irritation when applied to mucous surface; low percentage solutions necessary as compared with other similar preparations."

Silvol is supplied in powder (ounce bottles) and in 6-grain capsules (bottles of 50). The contents of two capsules make one-fourth ounce of a 10-per-cent. solution. Silvol Ointment (5-per-cent.), for application to regions where the use of an aqueous antiseptic solution is not feasible, is also offered. This ointment is marketed in long-nozzled collapsible tubes—two sizes, designated as large and small.

---

### RACHITIC CHILDREN.

The value of cod liver oil in rachitis has been so thoroughly demonstrated that there can scarcely be any question on the score of therapeutic efficiency, so the only problem arising in the use of cod liver oil in rachitis would be on the point of palatability, and if Cord. Ext. Ol. Morrhuae Comp. (Hagee) be adopted then this is at once settled. Cord. Ext. Ol. Morrhuae Comp. (Hagee) contains the essentials of the crude oil—the elements that give to the oil its well marked therapeutic and nutritive properties.

---

The widespread attention that has been roused during the past year on the subject of pyorrhea and its treatment with the ipecac alkaloids has included the physician as well as the dentist. Physicians in making diagnoses are becoming more and more impressed with the importance of seeking the point of infection in many pathological conditions that have stubbornly resisted treatment. The treatment of pyorrhea has been attended by improvement in a number of ailments which are now known to follow in the wake of gingival infections. Secondary conditions such as arthritis, neuritis and myalgias are often partially or entirely relieved after the removal of oral infections by proper instrumentation and ipecac medification.

There are three methods of administering the ipecac alkaloids—the local, subcutaneous, and oral. The less troublesome of these, obviously, is the oral treatment made possible by Alcresta Tablets of Ipecac, which are uncoated, disintegrate readily and yet cause no nausea. The alkaloids are liberated in the alkaline media of the intestines due to the fact that the ipecac alkaloids in these tablets are peculiarly combined with a form of hydrated aluminum silicate which does not release them in the stomach but readily liberates them in the alkaline intestinal juices. Eli Lilly & Company will be pleased to supply our readers with further information on the use of ipecac in pyorrhea and many kindred ailments having their source in infections about the mouth and teeth.

---

### AN ALTERATIVE OF LONG SERVICE.

It is mainly in chronic skin and glandular diseases that alteratives have found their distinct field of usefulness, for these are conditions aggravated and continued by impaired nutrition and elimination, in the correction of which alteratives show what potent remedial forces they are. Among the alteratives IODIA (Battle) has long enjoyed profesional favor and in this will be found a striking demonstration of its value, for no class of drugs is put to a more rigid test than alteratives, so its long continued use by physicians is the best evidence that it meets the demands made upon it. IODIA (Battle) will show its power in chronic skin diseases, glandular involvements and in other states indicating the corrective influence of an alterative agent. A distinct advantage offered by IODIA is that it may be continued over long periods without causing distress.

The Nervous Symptoms of Typhoid respond readily to

PASADYNE

(DANIEL'S CONCENTRATED TINCTURE OF PASSIFLORA INCARNATA.)

and you need not fear any depressing after-effects.

Reliable—Without Habit—Safe.

IT HAS NO CONCERN WITH THE HARRISON ACT.

SAMPLES AND LITERATURE SUPPLIED TO PHYSICIANS PAYING EXPRESS CHARGES

LABORATORY OF JOHN B. DANIEL, INC., ATLANTA, GEORGIA.

### A RARE REPUTATION.

A rare reputation among soothing and soporific agents has been earned by PASADYNE (Daniel)—the concentrated tincture of passiflora incarnata. This enviable reputation has been gained by PASADYNE (Daniel) because of its potency of therapeutic effect coupled with its marked freedom from disagreeable influences. Even in moderate dosage its tranquilizing power becomes manifest. A sample bottle may be had by addressing the laboratory of John B. Daniel, 34 Wall St., Atlanta, Ga.

---

Tissue resistance--that's the whole story. Following pneumonia or a severe Bronchitis, the patient drops into chronic invalidism or slowly climbs back to health. The deciding factor is tissue vitality. Possibly the damaged tissues may have a little recuperative power left—enough to make the climb, but why take a chance?

In Cord. Ext. Ol. Morrhuae Comp. (Hagee) you have a tissue food, a reconstructive of the highest order and through its supporting influence convalesence is lessened and return to health more certain.

Cord. Ext. Ol. Morrhuae Comp. (Hagee) possesses in addition to the foregoing virtues the advantage of palatability.

### THE ELIMINATION OF OPIUM'S UNTOWARD PHENOMENA.

Were it not for its several disagreeable features which are sufficiently weighty to make one hesitate before employing it, opium, of course, would be the ideal analgesic. Unfortunately, however, along with its analgesic effects, opium exerts those well known phenomena which tend to limit its usefulness as a pain-relieving agent.

But with the discovery of processes by which it is possible to eliminate the convulsive and narcotic principles of the drug, PAPINE (Battle) became possible, and with a wider therapeutic application than opium.

In the manufacture of PAPINE, the several objectionable qualities of opium have been eliminated, the finished product representing the analgesic and sedative properties only of this valuable drug.

In view of this, the superiority of PAPINE over opium and its alkaloids cannot be denied, for although offering to the patient the positive analgesic properties of opium it does not at the same time bind up his bowels or subject him to its other disagreeable effect. The utmost care is taken in the manufacture of PAPINE and it is fully believed that it offers every possible advantage over opium.

---

### DRESSINGS IN SUPPURATING WOUNDS.

The healing of suppurating wounds may be expedited in a marked degree by the use of ECTHOL (Battle). In addition to a germicidal influence it adds to cellular resistance, as a result of which the luxuriant germ growth becomes inhibited, until finally the purulent process becomes reduced to the point where the resistance of the involved tissues trusn the tide toward healthy granulation. Where such wounds are of more than ordinary size or severity, the internal administration of ECTHOL has proven the most useful adjunct to the local treatment.

### WHEN THE STOMACH IS TIRED OR LAZY.

The artificial digestives such as pepsin, pancreative papain, etc., have their place in modern therapy, but they should always be used with care and common sense. How often do we encounter patients who are continually dosing themselves with pepsin or some one of the artificial digestives after each meal? Ninety-nine times out of a hundred this is unwise and a positive harm. Instead, the process of digestion should be encouraged—the stomach urged to do its own work—for any remedy that will specifically stimulate these functions to nearer normal action will produce permanent benefits that can never come from pepsin. Seng, is such a remedy, with a well defined secernent action on the glands and mucus membranes of the stomach that enables it to restore and increase the functional activity of an organ that in the great majority of instances is only over tired or indolent.

---

The ampoule is a refinement in the administration of drugs hypodermically, and physicians have been quick to appreciate the superiority of this form of medication over that of preparing solutions from tablets. The ampoulse insures sterility of solution, accuracy of dosage and convenience in emergencies.

The ampoule often makes it possible to secure results that would not be attainable by oral administration; the effects are prompt, the dose is small, and there is usually less disturbance of the normal body functions.

Hermetically sealed ampoules prepared at the Lilly Laboratories under the strictest aseptic precautions, can be kept in a sterile condition indefinitely. Each Lilly ampoule is scratched at the constriction just above the body so that it may be broken easily and the contents withdrawn with the syringe. In addition to the outer box, each ampoule is packed in an individual carton, fully labeled. The physician may carry one or more loose in his medicine case or pocket safe from breakage.

### A NOTABLE GERMICIDE.

It is becoming more and more apparent, as time passes, that in Silvol we have a germicide of uncommon usefulness. Its field embraces practically all inflammations of mucous membranes. The indications for Silvol include conjunctivitis, corneal ulcer, trachoma, rhinitis, sinus infections, otitis media, pharyngitis, tonsillitis, laryngitis, gonorrhea, cystitis, posterior urethritis, vaginitis, cervical erosions, endometritis, etc., all infections, in short, in which a silver salt is applicable.

Silvol would appear to have a number of advantages over most of the other proteid-silver compounds. It is freely soluble in water. While an exceptionally powerful antiseptic, it is non-irritating in ordinary dilutions. Silvol solutions are not precipitated by proteids or alkaloids or any of the reagents that commonly affect other silver compounds in solution. They do not coagulate albumin or precipitate the chlorides when applied to living tissue.

In the treatment of acute inflammtaions of mucous membrane, Silvol may be used locally in solutions as strong as 50 per cent. with very little pain or irritation. In inflammatory affections of the ear, nose and throat it may be used in 5- to 40- per cent. solution, and for irrigating sinuses a 2- to 5-per cent. solution may be employed with benefit. For inflammatory conditions of the eye and conjunctival infection with pneumococci and staphylococci, a 10- to 40-per cent. solution may be applied with benefit three times a day. In acute gonorrhea, as an abortive measure, a 20-per cent. solution should be injected every three hours, while in the routine treatment the injection of a 5-per cent. solution three times a day is recommended.

### REPRINTS

Of Original Articles published in the Journal-Record of Medicine will be furnished at the following prices:

**500 Copies 50c per page.**

**1000 Copies 75c per page.**

When Reprints are desired it should be indicated on the copy.

**SAL HEPATICA**

**AN EFFERVESCING SALINE COMBINATION**

**LAXATIVE AND ELIMINANT**

BRISTOL-MYERS CO.
NEW YORK

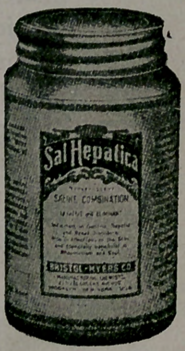

### "IN PARTICULAR CASES."

Therapeutic efficiency in the use of the bromides is [illegible] s dependent on the avoidance of untoward effects as on the attainment of maximum physiologic activity. For this reason Peacock's Bromides offer the most satisfactory bromide therapy, for not only does this happy combination of carefully selected bromide salts insure all the benefits the most active bromide preparation, but it does so with the great advantage that gastric disturbances and all tendencies to bromism are reduced to a minimum. This is why in "particular cases" so many physicians are in the habit of insisting on the use of Peacock's Bromides.

---

### NERVOUS IRRITABILITY FOLLOWING DEBAUCH.

For the relief of the nervous irritability following over indulgence in alcohol, PASADYNE (Daniel) will produce results of a highly gratifying character. These patients want cessation of the intense irritability, they want sleep, and in PASADYNE (Daniel) they may secure them. PASADYNE (Daniel) the concentrated tincture of passiflora incarnata is the ideal sedative. Effective and free from untoward results, it can be given without a feeling that evil effects may follow.

A sample bottle may be had by addressing the laboratory of John B. Daniel, 34 Wall Street, Atlanta, Ga.

---

### AN EASILY DIGESTED COD LIVER OIL PRODUCT.

The therapeutic value of a cod liver oil preparation depends upon the ease with which it is digested and assimilated. If it distresses the stomach and is not assimilated, its value as a therapeutic agent is nil. Thus the need of choosing a cod liver oil product that is well received by the stomach and is quickly assimilated. In Cord. Ext. Ol. Morrhuae Comp. (Hagee) these several requirements are met. In this cordial the essential principles of the plain oil are preserved unchanged, its disagreeable features (the grease) being eliminated. Cord. Ext. Ol. Morrhuae Comp. (Hagee) possesses every therapeutic virtue of the crude oil with the added advantage of palatability.

